# Embodied Displays of “Doing Thinking.” Epistemic and Interactive Functions of Thinking Displays in Children's Argumentative Activities

**DOI:** 10.3389/fpsyg.2021.636671

**Published:** 2021-02-18

**Authors:** Vivien Heller

**Affiliations:** Department of German Studies, School of Humanities and Cultural Studies, University of Wuppertal, Wuppertal, Germany

**Keywords:** thinking face, multimodal gestalts, posture, gaze, epistemic stance, argumentation, decision-making, conversation analysis

## Abstract

This study investigates moments in which one participant in an interaction embodies that he is “doing thinking,” a display that is commonly referred to as “thinking face. ” From an interactional perspective, it is assumed that embodied displays of “doing thinking” are a recurring social practice and serve interactive functions. While previous studies have examined thinking faces primarily in word searches and storytelling, the present study focuses on argumentative activities, in which children engage in processes of joint decision-making. The paper has two interrelated aims. The first aim is to describe how multiple modalities—beyond the face—are temporally coordinated to create multimodal gestalts of “doing thinking.” It is shown that thinking displays not only involve dynamic imaginative gaze but also stylized bodily postures. The second aim is to generate knowledge about the functions of thinking displays in children's argumentative activities. The analysis describes how both speakers and recipients use thinking displays in different turn positions and align them with verbal talk or silence. The data for this study comprise video recordings of decision-making processes in groups of older children. Drawing on a multimodal approach to situated interaction, it will be proposed that embodied displays of “doing thinking” provide a resource to shape participation frameworks, mark epistemic stances and create epistemic ecologies for collaborative reasoning. By investigating thinking displays in a particular conversational activity, the study sheds light on the diversity and context-sensitive functionality of thinking displays. It also contributes to recent research on children's collaborative reasoning as an embodied discursive practice.

## Introduction

Facial expressions are an integral part of face-to-face interaction and shape the way we interpret the actions and stances of our interlocutors. Research disagrees as to whether facial expressions represent a mere epiphenomenon of interaction or an interactive resource. They are thus conceptualized either as an externalization of emotional and cognitive states or as interactive resources. From these different perspectives, various facial expressions were described in more detail, including smiles (Ekman and Friesen, 1975; Kaukomaa et al., 2013), frowns (Ekman, 1979; Kaukomaa et al., 2014), and so-called thinking faces (Goodwin and Goodwin, 1986; Bavelas and Chovil, 2018). The latter typically entail that speakers withdraw their gaze; in addition, the gaze is not focused on persons or objects in the immediate surroundings but instead assumes a “middle-distance” look. Previous studies have shown that thinking faces are often used in word searches and storytelling. In their seminal study on word searches, Goodwin and Goodwin (1986) revealed that in those moments, when the speaker is hesitating and speaking, the thinking face serves as a visible display of his continued involvement in the joint activity, preventing the co-participants from taking the turn. This research demonstrates that thinking faces have important interactive functions. So far, however, little is known about the role thinking faces play in other discursive practices and whether they are used not only by speakers but also by listeners. It is also noticeable that previous research has focused exclusively on the face, arguably because it plays a prominent role in displays of “doing thinking.” However, such displays might involve other less prominent, but nevertheless relevant resources.

This paper addresses these issues and examines thinking faces in children's argumentative decision-making. Understanding argumentation as an interactively organized and embodied discursive practice (e.g., Mirivel, 2011; Jacquin, 2017; Heller, 2018) and drawing on a multimodal approach to situated interaction (Goodwin, 2000), the sequential analysis describes how both speakers and recipients combine various resources to create a complex multimodal gestalt that embodies “doing thinking.” It is proposed that what will be called *thinking postures* together with *imaginative gaze* and vivid eye movement are constitutive components of these displays. Furthermore, it is shown that these displays frequently occur in hypothetical scenarios where they are combined with lexical, morphological and syntactical markers of epistemic stance. In these conversational contexts, embodied displays of “doing thinking” serve not only interactive but also epistemic functions. It will be argued that they contribute to organizing thinking as public practice and to creating epistemic ecologies for collaborative reasoning. Such ecologies can be considered essential for establishing the “jointness of emerging decisions” (Stevanovic et al., 2017) in argumentative processes.

I begin by discussing previous research on facial expressions, thinking faces, and argumentation as an embodied discursive practice. Subsequently, I present the data and explain the analytical approach to the description of embodied displays. The analysis is divided into two parts. The first section examines the displays of speakers, the second the displays of recipients. A discussion of the findings concludes the paper.

## Theoretical Perspective

As yet, thinking displays were conceptualized as a facial expression. Accordingly, Goodwin and Goodwin (1986) coined the term “thinking face.” As an essential component of what Goffman calls “the personal front” (1963, p. 25), the face is an integral part of social interaction. Through facial movements participants show themselves “to be situationally present” (ibid., p. 27) and responsive to the obligations of their involvements with others. Compared to other body parts, the face is particularly mobile and flexible. As noted by Kidwell (2013, p. 104), these properties make it “an especially useful resource as both a stand-in for, and elaborator of, talk.”

Previous research on facial expressions can be categorized into two major strands of research that approach facial expressions as an externalization of inner emotional or cognitive states (e.g., Darwin, 1872; Ekman and Friesen, 1969; Ricci Bitti, 2014) or as interactive resources (e.g., Birdwhistell, 1970; Kendon, 1976; Chovil, 1991; Peräkylä and Ruusuvuori, 2006; Bavelas et al., 2014; Crivelli and Fridlund, 2018). While the former tends to focus on the individual, the latter investigates facial expressions as a social phenomenon.

Although Ekman and colleagues examine facial expressions both as emotional expressions and as conversational signals, their focus is on the face as “the primary site of affect displays” (Ekman and Friesen, 1969, p. 71), i.e., on the ways in which “internal” emotional states are expressed and recognized in and through the face. The assumption is that emotional expressions have inherent and stable meanings whereas conversational signals only emphasize, underline, and modulate verbal talk. According to Ekman, emotional expressions are spontaneous and occur early in ontogenesis. In contrast, conversational signals are used intentionally and acquired only after children have developed some “intentional language” (Ekman, 1979, p. 191). Methodically, Ekman has approached the study of facial expression either through detailed description of the muscles that are involved in producing a specific facial expression or through judgement tasks based on photographs. As Goodwin et al. (2012, p. 17) clearly show, this approach has considerable shortcomings: the face is examined in isolation from the speaker's body and the bodies of the co-participants; second, the “unfolding flow of an action in interaction” is ignored.

Interactional traditions conceive facial expressions as “visible acts of meaning” and examine the ways in which they “are part of the integrated message with words” (Bavelas and Chovil, 2000, p. 166). Emphasizing the functional similarities between facial expressions and gestures, Bavelas et al. (2014, p. 16-17) adopt Kendon's (2004, p. 310) notion of “facial gesture” to refer to “any configuration or movement of the face or head (including the eyes) that is synchronized with speech in both timing and meaning.” In addition, they apply Kendon's distinction between referential, interactive (or interpersonal), and pragmatic gestures to facial expressions. In a study on facial gestures in storytelling, Bavelas and Chovil (2018) observe that the majority of facial gestures serve pragmatic rather than referential functions.

While Bavelas and colleagues examine facial gestures by relating them to individual utterances, Peräkylä and Ruusuvuori (2006) investigate facial displays within the framework of Conversation Analysis. Inspired by the work on mutual monitoring and organization (Goodwin, 1980, 1981), they focus on the interplay and temporal organization of facial and other forms of expressions. Furthermore, they are interested in the role facial expressions play in different conversational activities, e.g., assessments (Peräkylä and Ruusuvuori, 2006) and storytelling (Ruusuvuori and Peräkylä, 2009; Peräkylä and Ruusuvuori, 2012). They show that facial expressions can project, accompany, and follow lexical elements that encode the speaker's stance and thus extend the boundaries of the spoken turn of talk. Furthermore, they examine how recipients respond to the speaker's facial stance displays and how they produce facial expressions themselves to shift the speaker's stance (Kaukomaa et al., 2015). This research demonstrates that facial expression is a highly flexible interactional resource that can be easily adapted to the contingencies of the activity-in-progress. Following this line of research, the present study analyzes embodied displays of “doing thinking” as social phenomena that are sequentially and interactively organized.

### The Thinking Face

The thinking face is one of several displays with which participants enact “doing thinking” and convey their stance toward what is being said. For instance, (facial) shrugs are used to display a distanced, less than committed stance (Streeck, 2011, p. 189f.) or to claim that something is obvious (Kendon, 2004; p. 275). Raised eyebrows (together with other facial actions) are reported to display disbelief, mock astonishment, or sophisticated skepticism (Ekman, 1979, p. 188f.). Frowns provide a resource to mark something as problematic and thus help preserve intersubjectivity in problematic conversational moments (Kaukomaa et al., 2014).

Thinking faces have occasionally been mentioned in research, though under different terms. Darwin (1872, p. 228f.) noted the “vacant expression of the eyes” that typically occurs “when a man is completely lost in thought.” He observes that the unfocused eyes can be accompanied by other movements or gestures, such as raising the hands to the forehead, mouth, or chin. Given the fact that Darwin did not have the opportunity to examine the temporal unfolding of interaction, it seems remarkable that he actually draws attention to a number of relevant components beyond facial expressions. Yet for him, this facial expression reflects an actual state of mind and is associated with processes of “abstraction” or “meditation” (ibid., p. 228).

Unlike Darwin, Goodwin and Goodwin (1986) conceptualize what they call “thinking face” not as the expression of an inner state, but as a sedimented and socially shared conversational resource. They observe that speakers who are involved in a word search withdraw their gaze and produce a characteristic, stereotypic thinking face. In such moments, the gaze is not focused on persons or objects in the immediate surroundings but instead assumes an “out of focus “middle-distance” look” (Goodwin, 1987, p. 117). Goodwin and Goodwin suggest that the thinking face is used as an interactional resource rather than being an adjustment to the cognitive demands that a word search entails: during a moment when the speaker is not speaking, the thinking face serves as a visible display of the speaker's continued involvement in the joint activity (storytelling). Through small changes in the facial expression and other resources such as fillers, pursing and slackening of the lips, opening or dropping the hand, and wh-questions such as “What the heck was it?”, the speaker visualizes distinct stages in his search for a word. In this way, the display works to prevent the co-participants from entering the unfinished turn. Furthermore, as speakers move through these stages, they can change the participation framework by resuming eye contact to solicit help from the recipients, thereby contextualizing the word search as “a visible activity that other participants not only recognize but can also participate in” (Goodwin and Goodwin, 1986, p. 52). This is why Bavelas et al. (2014) assume that thinking faces serve interactive functions as opposed to other pragmatic functions (i.e., modal, performative, parsing, cf. Kendon, 2004, p. 158f.). However, in another study on “remembering” as an interactional resource in storytelling, Goodwin (1987) conceptualizes thinking faces as displays of uncertainty. Thus, it seems that they can also serve modal functions, i.e., alter the frame in terms of which an utterance is to be interpreted.

Other studies examine thinking faces in elicited talk. Chovil (1991, 1997) studies the frequency with which different facial expressions occur in different activities (planning a meal, retelling a conflict, and a close-call experience). In her data, only speakers produce thinking faces. In what she calls “non-redundant” facial displays, thinking faces account for more than a quarter of all facial expressions. Furthermore, the multimodal composition of thinking faces varies slightly depending on the conversational context. In addition to withdrawing gaze and looking “thoughtful,” speakers sometimes also “lower eyebrows in a frown, or raise them while looking off in the distance, close their eyes, pull one side of their mouth back or twist their mouth to one side” (ibid., 182f). Building on a study by Clark and Fox Tree (2002), who describe “uh” and “um” as “collateral signals,” Bavelas and Chovil (2018) examine thinking faces in elicited telling. Thinking faces that occur at the beginning of the story are usually long. In addition, thinking faces are usually produced at transitions to other details or word searches. In most cases, they are introduced or accompanied by some form of verbal collateral signal; therefore, the authors suggest that the thinking face itself is a “collateral signal.”

In summary, it can be noted that thinking faces serve the recipient as recognizable displays that the speaker is currently involved in a word search or engaged in “remembering.” These observations are related to word searches and storytelling activities. Further functions mentioned by previous studies include the projection of new topics or thematic transitions and the display of epistemic uncertainty. To date, only speakers' thinking faces have been described, and only few studies have focused on the temporal unfolding of thinking faces in interaction. Addressing this gap, the present study describes how multiple modalities—beyond the face—are temporally coordinated to create multimodal gestalts (Mondada, 2014) of “doing thinking.” Furthermore, it investigates thinking displays in children's argumentative activities.

### Argumentation as an Embodied Discursive Practice

In face-to-face interaction, argumentative activities are a multimodal and multiparty field of activity. From the perspective of the sociology of knowledge and linguistic anthropology, arguing can be considered a sedimented discursive practice, i.e., as a socioculturally evolved procedural solution for recurrent communicative problems in a speech community (Bergmann and Luckmann, 1995; Hanks, 1996). The communicative problems argumentations are designed to overcome are the interactive management of divergent validity claims as well as the articulation and exploration of (potential) problems (Knoblauch, 1991; Antaki, 1994; Quasthoff et al., 2017; Arendt, 2019). Arguing is thus closely related to the co-construction of knowledge: it can be a vehicle for exploring proposals, negotiating divergent viewpoints, and making joint decisions (e.g., Stevanovic, 2012; Heller, 2018). At the same time, arguing enables participants to constitute social orders and negotiate identities (e.g., Goodwin and Goodwin, 1987; Danby and Theobald, 2014).

Recent research suggests that epistemic stancetaking (e.g., Kärkkäinen, 2006; Heritage, 2012) provides a crucial resource for argumentative activities (Keisanen, 2007; Heller, 2018; Kreuz and Luginbühl, 2020; Morek, 2020). Here, the display of a tentative, uncertain or determined stance is especially consequential not only for shaping local participant frameworks but also for the larger framing of the activity as persuasive and competitive or as exploratory and collaborative (Sterponi, 2009; Ehlich, 2014; Bova and Arcidiacono, 2015; Heller, 2018; Mundwiler and Kreuz, 2018; Hannken-Illjes and Bose, 2019). These frames entail different epistemic orders that differ in the degree to which the jointness of an emerging decision (Stevanovic et al., 2017) is established.

In addition to epistemic stance displays, embodied resources are also vital for decision-making processes. Stevanovic et al. show that body-sway patterns and pitch register provide important resources for interpersonal coordination in joint decision-making. Hannken-Illjes and Bose (2019) show how children use the synchronization of bodily actions and paraverbal resources such as loudness to frame their argumentation as cooperative whereas what they call “agonistic situations” exhibit a rather arhythmical or discontinuous coordination. Manual actions and gestures are other important resources to make “embodied arguments” (Mirivel, 2011). For example, reciprocal palm up open hand gestures (Kendon, 2004; Müller, 2004; Streeck, 2007) serve as publicly visible resources to embody the give-and-take of arguments (Schönfelder and Heller, 2019), thus facilitating the orderly production of contiguous responses. In a similar way, interlocutors use gestures as a device to facilitate understanding by segmenting structural parts of their arguments (Jacquin, 2017). These studies indicate that arguing is an embodied discursive practice. The present paper investigates the thinking face as a potential resource for interpersonal coordination in children's argumentative activities.

In a previous study on embodied resources in children's argumentative decision-making, Heller (2018) examined a sequence in which the thinking face served as a framing device for organizational problems on various interactional planes. First, by projecting that the performer of the thinking face is going to claim the floor, the display facilitates the organization of turn-taking. The publicly visible performance mobilizes the recipients‘visual attention. The latter shift their gaze to the speaker and refrain from taking the turn. Second, by giving the audience a clue to what kind of action the turn will be doing, the thinking display provides an important device for action formation. Together with lexical and morphological resources (e.g., verba dicendi, subjunctive mood), it serves to display a thoughtful stance and thus instructs the recipients to expect the ensuing action to encompass a disclosure of the incipient speaker's thoughts, i.e., a proposal and the “thoughts behind it.” Third, with regard to the larger activity, the placement at the beginning of the sequence projects how the speaker conceptualizes the nature of the joint project—argumentative decision-making—as one that involves thorough thought. The thinking face thus also helps to frame the activity as collaborative reasoning^1^. The study indicates that thinking faces can fulfill several functions for the framing of argumentative decision-making and the coordination between the participants. However, it is based on a single instance. The present study therefore examines interactional and epistemic functions of embodied displays of “doing thinking” in a systematic approach.

## Materials and Methods

### Participants and Data

The data for this study come from a larger corpus of video recordings of 90 monolingual and multilingual children aged 7–13 years. All children attended inclusive classes in primary and secondary schools, located in different socio-economic milieus in Germany. For the present study, only 10 groups of 32 older children (aged 10–13 years) were selected. Within the school setting, groups of three to five children were video recorded as they dealt with different problems. One of these problems concerned a fictitious shipwreck and required the children to make a joint decision on essential survival items (for a similar setting see Kreuz and Luginbühl, 2020). Another task entailed a moral dilemma that arises in the course of an attempt to cheat in a painting competition. Both problems provoked argumentative activities in the groups. For each task, there was a handout with a graphic illustration on the table. The children sat in a semicircle around the table and pulled the handout toward them, pushed it into the middle and pointed to individual illustrations. To minimize disturbance of the groups, only one camera was used. Because the children sat in a semicircle, occurrences of thinking displays were usually easy to identify, but not always visible in detail. The analysis is therefore limited to children at whom the camera was directed frontally. The video recordings used for the present study comprise 58 min in total.

### Analytical Approach

Videos were pre-coded for all occurrences of thinking faces. Each instance was coded to indicate whether it was produced by the speaker or the recipient. After the analysis of different cases, the data were reviewed a second time. This way, a total of 29 thinking displays of speakers and 28 of recipients were identified. All occurrences were analyzed in detail, using a multimodal approach to situated interaction (Goodwin, 2000; Streeck et al., 2011; Heath and Luff, 2013). By examining how participants themselves orient to each other's actions sequentially, the analysis reconstructs how they use and interpret thinking faces and accomplish particular activities. In analyzing the total of 57 occurrences, five practices of embodying “doing thinking” emerged. The following section presents a prototypical example for each practice.

The present paper has two interrelated aims. Given that embodied displays of thinking have received only little empirical attention, the first aim is to describe how multiple modalities—beyond the face—are temporally coordinated to create various multimodal gestalts of “doing thinking.” A second aim is to generate knowledge about the interactive and epistemic functions of thinking faces in children's argumentative activities.

The detailed description of embodied displays of “doing thinking” tries to reconstruct what is visible to the interlocutor as a whole. Since not only facial expressions but also other modalities are involved, I refer to these displays not as thinking faces, but as *embodied displays of “doing thinking*.” They are best conceived as multimodal gestalts (Mondada, 2014, p. 139), i.e., a “web of resources formatting an action.” Accordingly, the analytic orientation is on the multiple modalities that contribute to embodied displays of thinking, e.g., typical body postures, particular gaze practices, silence or specific linguistic resources. Since in the data thinking displays were closely associated with the use of (other) epistemic stance markers, special attention is paid to lexical (e.g., modal adverbs and particles) and morphological (e.g., subjunctive) markers of epistemic modality as well as syntactical formatting (e.g., conditional constructions). The focus is on how these resources are temporally coordinated not only for the publicly visible display of “doing thinking,” but also for the organization of turn-taking, the shaping of embodied participation frameworks (Goodwin, 2003), the participants' positionings, and the framing of the larger activity.

The transcription follows the GAT 2 conventions proposed by Selting et al. (2011). The multimodal annotation was adapted from Mondada (2018). The description of facial and other bodily actions is informed by the emic approach and based on easily recognizable colloquial descriptions (Birdwhistell, 1970; Peräkylä and Ruusuvuori, 2006). To represent relevant bodily actions and action components, stills were extracted from the videos and temporally aligned with the emerging verbal utterance. For reasons of anonymization, the stills had to be converted into drawings^2^ that capture the most relevant aspects of bodily behavior. Bold print of single words or syllables marks the exact point in time when the frame grab was taken. Since the analytical focus is not on gesture, I do not transcribe different gesture phases to ease the readability of the transcript.

## Analysis: Various Embodied Displays of “Doing Thinking” in Argumentations

This section reconstructs different practices of embodying “doing thinking.” The thinking displays in the data varied in their multimodal gestalts, their turn position, and their alignment with verbal talk and thus serve different interactive and epistemic functions. Furthermore, both speakers and recipients have produced them. Speakers' practices entail embodiments of (1) envisioning and embodying a hypothetical scenario, (2) thoughtful searching for (a part of) an argument, and (3) presenting a position as well-reasoned and thought-through. Recipients' practices comprise embodiments of (4) co-imagining and exploring a scenario described by the current speaker, and (5) independent and critical thinking. The next section describes speakers' practices of using thinking displays.

### Speaker Displays of “Doing Thinking”

Speaker displays of doing thinking occur both in single-unit and multi-unit turns (i.e., “big packages,” cf. Sacks and Jefferson, 1992: II). When they are produced within multi-unit turns, they are often part of developing a hypothetical scenario and involve solitary or joint origo displacements (Bühler, [1934] 1999). In these contexts, they serve to establish a more or less shared responsibility for developing an argument. In contrast, their use in single-unit turns such as “simple” proposals or statements of opinion does not involve a displacement of the origo. In these contexts, thinking displays are used to present a position as already being well-reasoned and determined.

#### Envisioning and Exploring a Hypothetical Scenario

In multi-unit turns, thinking displays can take the form of an extended performance that accompanies speech. This type of temporal alignment implies that the audience participates in the development of a hypothetical scenario from the outset and that speaker and listener share the epistemic responsibility for its exploration. Extract 1 is taken from a group that consists of four children, Anna, Jona, Marko, and Sara. Only Jona and Sara are involved in the following sequence. Jona and Sara first disagreed on the question as to whether the tent is essential for survival. Then Jona establishes an obligation to provide reasons (Heller, 2014). As part of her reasoning, Sara produces a longer-lasting thinking display.

**Extract 1:**
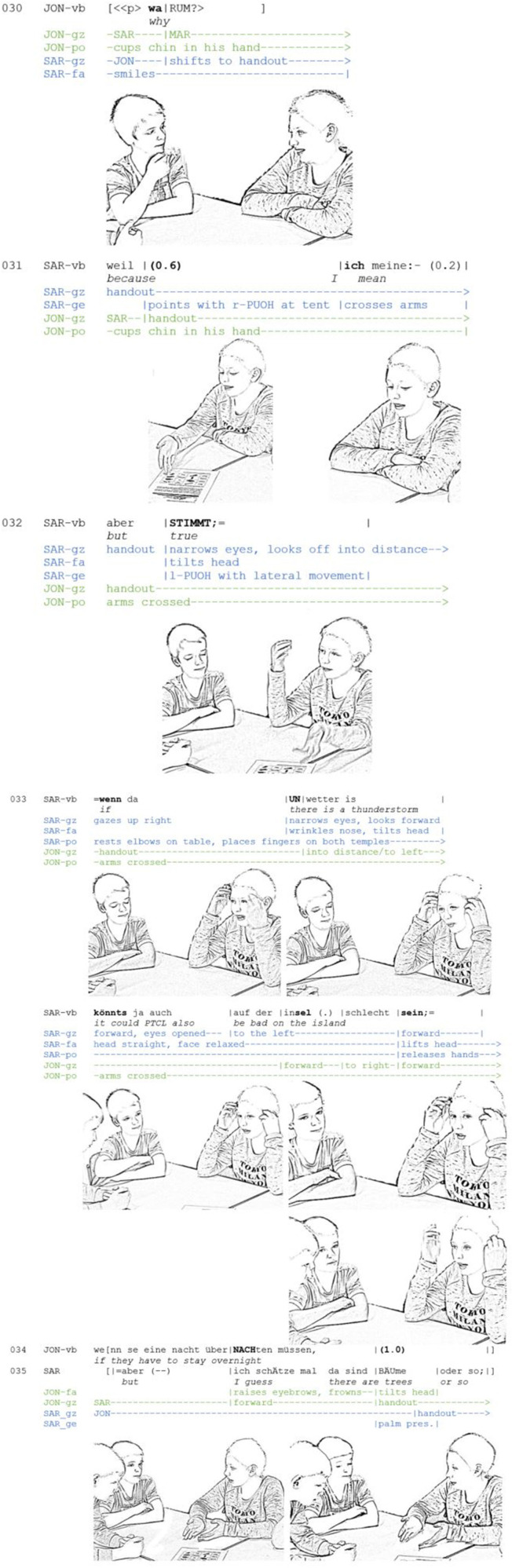
AE_G10_FB_30-34 (JON: Jona, SAR: Sara).

While Jona challenges Sara's position (line 30: “why?”), he cups his chin in his hand, thus assuming a thinking posture (see below) and conveying a thoughtful stance. Looking at each other, he and Sara establish a facing-formation (henceforth: F-formation, Kendon, 1990), entailing that participants “orient their bodies in such a way that each of them has an easy, direct, and equal access to each other's transactional segment” (including mutual gaze; Kendon, 1990, p. 239). When Sara begins to provide a reason, her pointing to the handout also shows that she is engaged with the objects and participants in the here-and-now (line 31). Overlapping with “I mean:-” she crosses her arms and leans back, thus gradually moving out of the F-formation. After that, she abandons her justification and concedes “but true.” At the same time she tilts her head, looks off into the distance, with her eyes narrowed and moves her open hand sidewards (“palm lateral,” cf. Kendon, 2004, p. 275). Note that she changes from the right to the left hand. According to Müller (2004, p. 249), such antagonistic lateral movements of the open hand add the idea of “cutting” to the core meaning of the so-called “palm up open hand gestures” (cf. Kendon, 2004; Streeck, 2007). Here, the gesture conveys that the speaker's original position is no longer pursued. Together with the other resources, the gesture thus indexes a change of mind. This *embodied change of mind* serves as a transition into the display of “doing thinking.” Resting the right elbow on the table, the speaker moves into an inflexible body posture.

The thinking face is produced in the context of a hypothetical scenario, which serves to explore the implications of a proposed action. The following resources are progressively assembled and combined to embody the process of *envisioning and exploring a hypothetical scenario:* first, the conditional “if” is temporally coordinated with another change in the bodily posture. The speaker rests the second elbow on the table and places her fingers on her temples, assuming an inflexible posture. In this posture, head, and hands are fixed, signaling that neither gestures nor head movements are to be expected for the duration of this bodily configuration. Furthermore, the posture entails that the speaker touches herself. As opposed to social touch that allows us to engage with others (cf. Goodwin, 2017), self-touch implies that the individual gets entangled in the haptic-kinetic perception of her own body and shields herself from other stimuli. This kind of posture can be considered stylized; similar postures are known, for instance, in arts (e.g., “The Thinker” by Rodin) and are called *thinking posture* here. Assuming this posture, the speaker creates a marked contrast between speaking and silence as well as movement and immobility, indicating that she is temporarily absorbed by her own thoughts. She thus brings about a change in her involvement with others (Kendon, 1990, p. 187). The posture is combined with looking up into an “empty space,” as if the idea were to be found on the ceiling (cf. Ekman, 1979, p. 186). This gaze withdrawal has the result that the F-formation is temporarily suspended. Instead, the speaker embodies a change in the direction of her attention: she indicates to her co-participants that she no longer perceives the external surroundings, but rather directs her attention inwards, toward a world of thought, in which she first needs to make up her mind before she can share her idea with her co-participants. Since the gaze is not focused on objects or persons in the here-and-now but instead on entities that only exist in the speaker's mind, I refer to this gaze behavior as *imaginative gaze*. The latter is interpreted by the participants with respect to the ongoing activity. This implies that this gaze practice assumes an interactional function (Rossano, 2012, 2013) and informs the participants' mutual understanding.

In her description of the scenario, Sara demarcates condition and consequence through observable changes in the emerging multimodal gestalt. The condition “there is a thunderstorm” (line 33) is accompanied by a display that conveys that the speaker is envisioning the scene in the moment of her own description. The head is tilted, the nose wrinkled and the eyes are narrowed and looking forward. In this way, the process of “zooming in” or “focusing on” a virtual image that gradually emerges before her mind's eye is embodied. In contrast, the hypothetical consequence “it could PTCL also be bad on the island” is temporally aligned with a relaxation of the face. At the same time, the head returns to an upright position; the eyes are opened and wandering to the left, conveying the impression that the scene is now clearly visible and explored in more detail. On the verbal level, the untranslatable German modal particle “ja” implies that what is just being said can be assumed to be shared knowledge (Reineke, 2016, p. 96). Altogether, the alignment and juxtaposition of these resources embody a change of state (Heritage, 1984) from an incomplete to a complete image, from an uncertain to a certain stance. While the thinking posture with the facial self-touch remains stable and thus functions as a bracket (Scheflen, 1965) for the protasis and apodosis, the changes in the facial expression and the wandering eyes embody two processes, envisioning and exploring a scenario. The whole process is completed when the speaker reaches the turn-final word “sein.” In this moment, she lets go of her temples and shifts her gaze to the front.

I would like to argue that the thinking posture and the imaginative gaze serve to indicate that the current speaker is “stepping out” of the here-and-now and displaces her origo, i.e., the “here-now-I system of subjective orientation” (Bühler, [1934] 1999, p. 117), to an imaginary space. It is important to note that this displacement is not accomplished privately but as a publicly visible performance. The temporal organization of the multimodal resources ensures that the recipients can likewise displace their origo. Through the simultaneity of verbal description and embodied imagination, they are enabled to jointly imagine the hypothetical scene. This can be clearly evidenced by the recipient's behavior: resting his crossed arms on the table, Jona signals that he assumes the role of the listener and will not interfere (line 32). When Sara begins to sketch the condition (“thunderstorm”), he lifts his gaze from the handout and starts to look into the distance. While Sara goes on to draw a consequence, he first continues to look to the empty space in front of him and then shifts his gaze to the right and back (line 33), conveying that he, just like Sara, is envisioning and considering the scenario from different sides. By refraining from looking at Sara, he demonstrates that he is not simply agreeing with her argument but responsibly exploring the scenario on his own. The recipient's reciprocation of the thinking face shows that the participants have established a participation framework for *joint imagination* (Stukenbrock, 2017; Heller, 2019; Kinalzik and Heller, 2020).

In this way, the argumentation is staged as not yet completed or unchangeable, but as being in the process of formation. Furthermore, the speaker enables the recipients to participate in “*the gradual production of thoughts whilst speaking”* (Kleist, 1805) and therefore invites them to explore the imagined scenario together with her. As fellow thinkers they *share the epistemic responsibility* (Stivers et al., 2011) for examining a hypothetical scenario. This is also underlined by the fact that Jona elaborates the scenario in his next turn: “if they have to stay overnight” (line 34). This co-construction (Kreuz and Luginbühl, 2020) further supports the reasoning and turns it into a shared argument. In comparison to the second example, the speech-accompanying embodiment of thinking has the effect that the listeners are involved in the process of imagination from the beginning and thus participate more intensely in developing the argument.

#### Thoughtful Searching for (a Part of) an Argument

In the data, thinking displays also occur when the speaker is searching for (a part of) an argument. In these cases, the thinking displays only last for the duration of the search. They also differ in terms of their multimodal gestalts and their coordination with speech from the displays described in the previous section.

Extract 2 is taken from a discussion about the moral dilemma. While Jona suggested that Tom should only talk to his parents about Marie's cheating (Tom and Marie both belong to a fictional scenario) at the painting contest and leave it up to them to decide whether to inform the teacher, Sara proposed that Tom himself should take action on the cheater. After Jona has abandoned his original position and supported Sara's proposal, Sara develops a justification for the now shared position. In the course of formulating her justification, Sara hesitates and engages in an embodied search for the second part of her argument.

**Extract 2:**
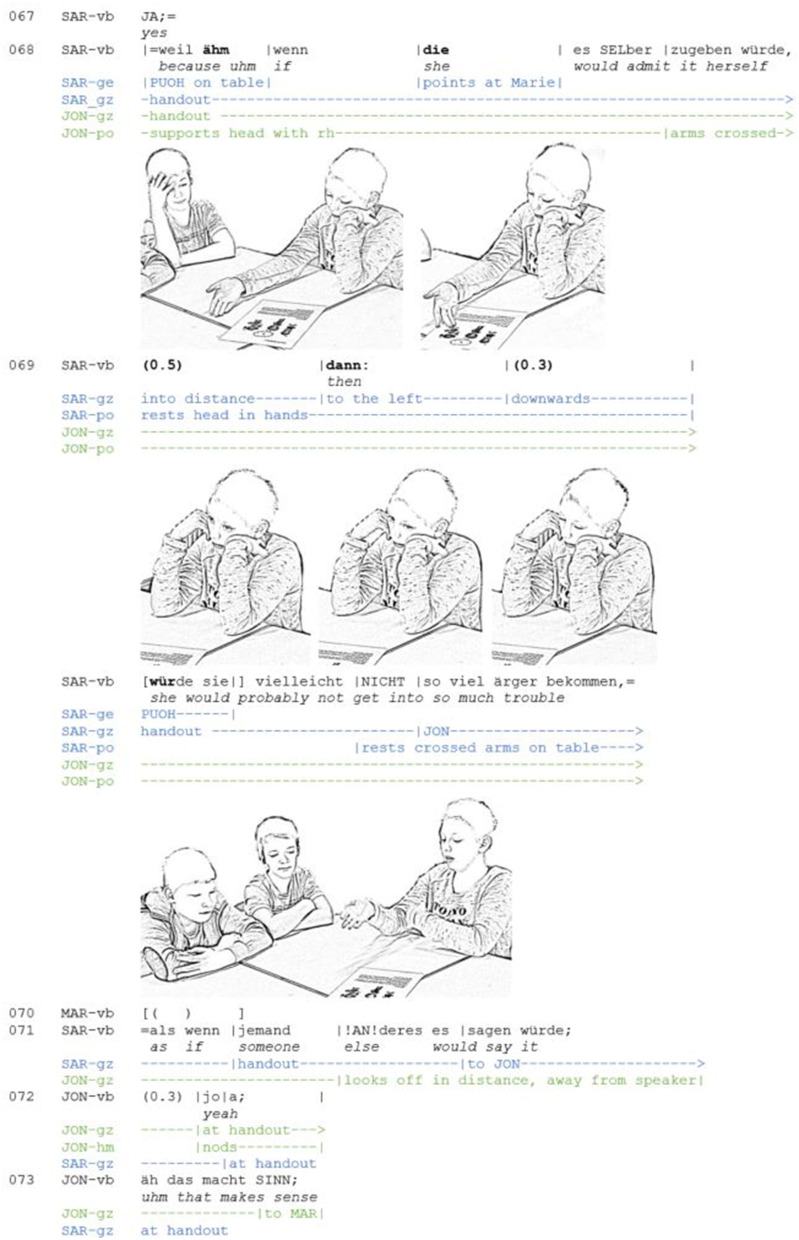
AM_G10_FB_67-73 (JON: Jona, MAR: Marko, SAR: Sara).

Sara projects her argumentation through verbal and bodily means. The causal connective *because* is temporally aligned with moving the open hand palm up toward the table. By extending the open hand into the participants' interactional space the speaker enacts the idea of “giving” or presenting an “abstract discursive object” (Müller, 2004, p. 233; also cf. Kendon, 2004, p. 264)—here: a reason –, and offers it for joint inspection. Placed at the beginning of a new unit of her multi-unit turn, the combined resources thus foreshadow the pragmatic function (cf. Streeck, 2009, p. 171) of “providing a reason.” In the present case, the reason includes a hypothetical scenario, which is marked by the conditional “if” (line 68) and the subjunctive. In this way, the speaker not only projects the type of action but also indicates her epistemic stance: what she is going to say has a hypothetical and tentative status. Sara uses the hypothetical scenario to play through the consequences of her proposal (getting Marie to confess her deceit). As she formulates the protasis (“if she would admit it herself,”), her gaze is focused on the handout, and with a pointing gesture, she disambiguates the reference of “she.” The recipient, Jona, follows her gesture. Thus, both participants' visual attention is oriented toward an object in their immediate here-and-now (Bühler, [1934] 1999). After the condition has been verbalized, two pauses and the lengthened “then:” indicate a hesitation. In the ensuing pause, Sara withdraws her gaze from the handout and shifts it to an empty space. Such gaze withdrawal while searching for a word has frequently been noted (Kendon, 1967; p. 38; Goodwin and Goodwin, 1986; Goodwin, 1987; Weiß and Auer, 2016). In addition to the imaginative gaze, Sara also retracts her arm and supports her head in her hands. This thinking posture emphasizes that the speaker is temporarily absorbed by her own thoughts or temporarily “away” (Goffman, 1963, p. 69)—engaged in solitary thinking and not ready to interact. In this way, it also serves to create an embodied participation framework for “forming an opinion before speaking.”

Decisive for the embodiment of *searching for (a part of) an argument* is the dynamic of the inwardly directed gaze, which is particularly prominent against the background of the immobility of the body. At first, the gaze is directed forward, then moved to the left and finally downwards, with the eyes almost closed. The eye movements are temporally coordinated with verbal resources or with their absence. The gaze forward coincides with the first pause; the wandering of the eyes to the right is aligned with “then:” i.e., with the verbal resource prefacing the consequence or apodosis. The downward oriented gaze, which also concludes the eye wandering, occurs in the second pause. This temporally aligned and dynamical wandering of the gaze embodies that a thinking process is currently taking place, involving that the speaker “sees” something with her mind's eye. While the forward gaze creates the impression that the speaker is searching for or developing an idea, the wandering of the eyes to the left, temporally coordinated with “then,” conveys that the idea is being advanced. The downward gaze, toward the handout on the table, where the eye movement halts, embodies that the line of thought is now so far developed that it can be shared with the co-participants. It also marks a transition, indicating that the attention is now turned away from the inner world and directed toward the world of discourse. At this moment, Sara releases her posture and performs another forward-gesturing palm presentation gesture to signal that the second part of her scenario, the consequence, is now going to be formulated. Simultaneously, she reorients her gaze to the co-participants and objects within the here-and-now.

Like the embodied envisioning and exploring of a hypothetical scenario, the thoughtful search involves an origo displacement. However, the displacement differs in one important point. Like in Extract 1, the freezing of bodily movements and the reorientation of the gaze serve to indicate that the current speaker is “stepping out” of the here-and-now and temporarily directs her attention to a world of thought that is only accessible to her. The publicly visible performance of “doing thinking,” embodied through the dynamically wandering gaze, instructs the recipients to interpret the speaker's displacement as a phenomenon that is nevertheless related to the current argumentative activity and therefore prevents them from taking the turn. In contrast to Extract 1, the speaker first performs a *solitary displacement*. The placement of the thinking display during the pauses (line 69) ensures that initially only she alone is able to “see” the imagined consequences. Only when she verbally formulates the apodosis, the recipients are enabled to co-imagine the scenario. By granting the recipients only delayed epistemic access, the speaker presents the consequence she has drawn as independently tested and weighed, thus positioning herself as a responsible thinker. At the same time, the display of uncertainty (the adverb “perhaps” in the apodosis) conveys that the co-participants have the right and responsibility to be involved in the decision-making process. In this way, the speaker balances the relationship between individual and shared epistemic responsibility. It can be observed that the recipients align with the speaker's embodied search: only when the argument is completed, Jona produces a thinking face himself (line 72), and then agrees with the argument (line 73).

In summary, it can be said that the thinking face here fulfills a similar function as in the word searches examined by Goodwin and Goodwin (1986). The main difference is that the search does not only cover one word, but a component of an argument. The fact that embodied searches are also successful in argumentative activities is remarkable, since proponents and opponents often compete for the turn. The framing of the argumentation and the sequential position in which the embodied search occurs is revealing in this respect. Sara initiates the search for a not yet available reason after an agreement has already been reached. Furthermore, for the most part, the argumentative activity is not framed as competitive but as collaborative (Heller, 2018). The search for a justification that is now taking place is not only made possible by this framing, but at the same time reflexively maintains it.

#### Presenting One's Position as Well-Reasoned and Thought-Through

A number of thinking displays are found in rather short turns, in which the speakers present their position without further supporting it. In these cases, embodied displays serve to present one's position as well-reasoned and already thought-through. Extract 3 represents the very beginning of the argumentative activity. After Sara has initiated the discussion (line 24: “so”), all parties involved allow a pause to arise (line 25), before Jona takes a stand and produces an accompanying thinking display (line 26). In contrast to the first two examples, his display does not evoke an ongoing thinking process but rather indicates that the process is already completed.

**Extract 3:**
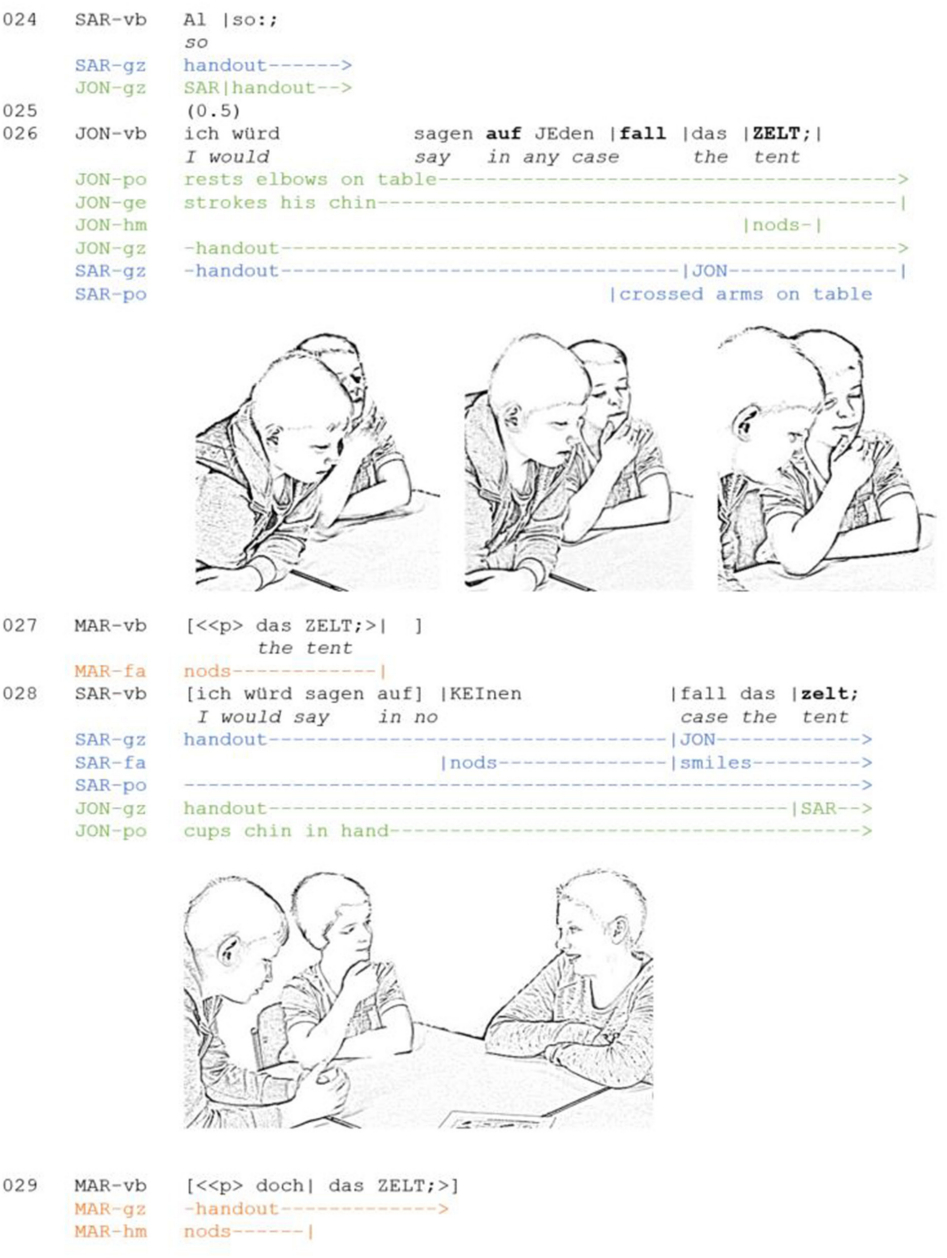
AE_G10_FB_24-28 (JON: Jonah, MAR: Marko, SAR: Sara).

Jona does not simply state his position, but projects it metadiscursively with an epistemic preface (Heller, 2018), using a verbum dicendi and the subjunctive (“I would say”). The preface serves to explicate the pragmatic function of “taking a position” and frames the contribution as a proposal. This means that the proposed decision is presented as one conceivable among possible others and thus as contingent upon the recipients' approval (Sidnell, 2012; Stevanovic, 2012). However, this tentative stance is modified by further epistemic markings in the course of the utterance (see below).

Temporally aligned with the preface, the speaker adopts an inflexible body posture. He rests his elbows on the table and cups his chin in his hands. At the same time his gaze is drawn away from the persons in his immediate interactional space and shifted toward the handout. Additionally, he starts to stroke his chin. Again, this form of self-touch can be considered to be a stylized thinking posture. Against the background of the inflexible body, the self-directed movement serves to mobilize the co-participants' (visual) attention, as can be seen in Sara's reaction: she shifts her gaze to Jona (line 26). Note that in this example the speaker does not shift his gaze while speaking (as in Extract 1). By keeping his visual focus on the handout, the speaker indicates that he is not displacing his origo to an imagined scene in order search or develop an argument. Instead, the constant focus of the eyes contributes to the impression that the speaker has already reached a decision. This is consequential for the epistemic order and will prove to be a major difference to the previous two examples.

In addition, the sequential placement of the thinking display is important: by adopting the thinking posture in turn-initial position and maintaining it throughout the turn's production, the speaker conveys that his or her position has already been thoroughly thought out and does not require further elaboration by the other participants. The speaker thus asserts epistemic primacy (Heritage, 2012). This is also emphasized through the epistemic idiom “in any case” and the nodding which concludes the embodied display. Both resources are used to express epistemic certainty and *present the proposal as well-founded* and not requiring further justification. By making only an agreement of the other participants relevant, they steer into closing the discussion of the item in question. In their subsequent turns, the co-participants refer directly to the position expressed by Jona. While Marc agrees with the opinion (line 27), Sara establishes a playful dissent by means of a format-tied response (Goodwin and Goodwin, 1987), which is accompanied by smile and mutual gaze (line 28). This friendly challenge to the speaker's pre-determined stance (Heller, 2018, p. 285) shows that such embodied epistemic positionings are not necessarily “successful” and may result in dissent and a rearrangement of the epistemic order.

To summarize, the thinking posture is adopted at the beginning of the turn and maintained for the time of its production. The multimodal gestalt of the display is mainly based on the thinking posture, steady gaze to the handout and the nodding of the head. Verbal resources entail an epistemic preface and markers of epistemic certainty. All these resources serve to present a position as already thought-through. The speaker indicates that he is no longer in the process of searching or developing ideas but has already come to a decision.

The embodied displays described in this section typically occur in short argumentative turns, in which a position is stated without further argumentative support. In contrast to the first two examples, the thinking displays occur in single-unit turns and are temporally organized in ways that do not involve the recipients in the development of an argument.

#### Summary

The analyses showed that “doing thinking” was organized as a public practice: the embodied displays of “doing thinking” involve multiple modalities. The components of the different multimodal gestalts are summarized in Table 1.

**Table 1:** Speakers' embodied displays of “doing thinking.”

**Envisioning and exploring a hypothetical scenario**	thinking posture	imaginative gaze and focusing, wandering of the eyes	accompanying speech (in a multi-unit turn that develops a hypothetical scenario)	joint imagination, shared epistemic responsibility
**Thoughtful searching for (a part of) an argument**	thinking posture	imaginative gaze and wandering of the eyes	during hesitation (in a multi-unit turn that develops a hypothetical scenario)	first solitary, then joint imagination, independent and shared epistemic responsibility
**Presenting one's position as well-reasoned and thought-through**	thinking posture	steady gaze to the handout	accompanying speech (in a single-unit turn in which a position is stated)	no displacement/imagination, epistemic primacy

Each of the multimodal gestalts entailed a stylized *thinking posture*. Although they took different forms—supporting the head or chin in the hand, grasping the temples–, they had in common that the body assumed a rather inflexible posture, while the eyes moved vividly. The analysis of the entire corpus shows that the type of posture is not decisive for the functions the thinking display fulfills. The functions rather depend on the activity of the eyes, the coordination with speech or silence and the sequential placement.

Two displays were characterized by *imaginative gaze* and eye movements that evoked a progress in thought. When imaginative gaze was coordinated with silence, it enacted a solitary displacement; when it accompanied the verbal description of the scenario, it had the effect of involving the listeners in the process of imagination. The absence of the imaginative gaze served to convey that the speaker was no longer in the process of searching or developing ideas but had already come to a decision. The different uses of thinking displays were consequential for the epistemic ecology of the activity in that they constituted an independent or shared epistemic responsibility for argumentative decision-making.

### Recipient Displays of “Doing Thinking”

This section examines recipient displays of “doing thinking” in multi-unit turns. With regard to displays of emotion, Kaukomaa et al. (2015) have demonstrated that the recipients' facial expressions not only display understanding of what is said but may perform systematic operations on the speaker's turn and the emerging activity. In the present data, recipients use thinking displays to demonstrate their alignment with the ongoing activity. In addition, they signal either agreement or disagreement, or an exploratory or critical stance. In this way, they provide an ongoing feedback not only for the current speaker, but also for the other co-participants. Thus, displays of “doing thinking” are a resource to shape the emerging participation framework while listening.

#### Embodying Independent and Critical Thinking

In the data, recipients were found to use embodied thinking displays to embody a critical stance and project that they are going to claim the floor. Extract 4 stems from a group of five children; only three of them are involved in the following sequence: Deana, Yeliz, and Zarif. Several proposals are made on how to deal with the cheating in the painting competition. When Deana makes an alternative proposal, Zarif visually displays that he takes an independent and critical stance on it.

**Extract 4:**
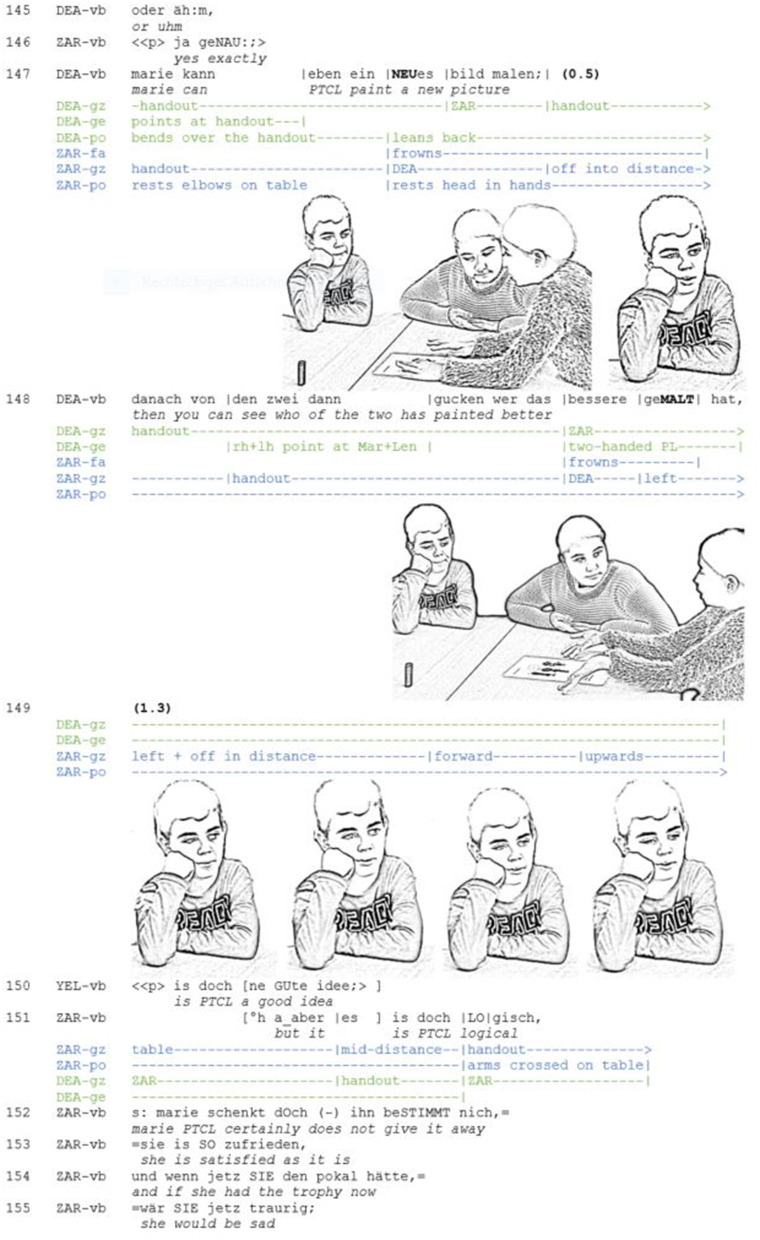
AM_G11_S_145-155 (DEA: Deana, YEL: Yeliz, ZAR: Zarif).

Shortly after Deana has projected an alternative proposal (line 145), Zarif agrees with a suggestion made by another participant (line 146). When Deana goes on to formulate her idea (line 147) and arrives at the semantic core element (“new picture”), Zarif does multiple things at once: he assumes a thinking posture, with his elbows rested on the table and his head supported by his right hand. Additionally, he frowns. Together with the thinking posture, the frown serves as a resource for the recipient to mark an element of the speaker's utterance as problematic (for speaker frowns see Kaukomaa et al., 2014). Looking at Deana, who shortly afterwards establishes mutual gaze, Zarif checks whether Deana notices his display. Establishing mutual gaze with Zarif while speaking, Deana is in fact able to perceive visually that Zarif is not only listening carefully, but also displaying a critical stance toward her proposal. At the end of the unit of the multi-unit turn, both participants dissolve eye contact. By withdrawing his gaze from the objects and participants in the interactional space and looking off into the distance, Zarif demonstrates to the other parties that he thinks independently about the proposal (Stevanovic, 2012) and forms his own opinion. In this way, he positions himself as a responsible and critical thinker. At the same time, Zarif demonstrates that he listens to Deana attentively: as the latter continues her turn (line 148) and disambiguates the reference with deictic gestures to the fictitious protagonists, Zarif's gaze follows Deana's hand. Toward the end of the turn, i.e., at the transition place, Deana and Zarif again establish mutual gaze. In this moment of mutual perception, Zarif frowns again and also presses his lips together, thus indicating stronger doubt. Simultaneously, Deana produces a two-handed palm lateral gesture, with which she declares the discursive object to be obvious (Kendon, 2004, p. 275f.; Müller, 2004, p. 243f.). Without speaking, Zarif has thus projected a dissent.

In the ensuing pause (line 149), Zarif transforms the frown into a prolonged display of “doing thinking” with which he projects, among other things, that he going to take the turn. The trajectory of facial expressions thus enables a smooth transition from the role of the recipient to that of the speaker. Furthermore, they serve as a pre-element for the ensuing disagreement (Pomerantz, 1984) and thus project that type of next action is to be expected. Maintaining the thinking posture, Zarif looks off in the distance, thus indicating a change in the direction of his attention. Then his imaginative gaze describes a circle. The thinking posture and the wandering of the eyes yield a multimodal gestalt that embodies the recipient's displacement to the scene previously described by the speaker. In contrast to the next example, the embodied displacement is solely performed by the recipient. Initiating the embodied display only at the end of the speaker's turn and maintaining it throughout the pause, the speaker demonstrates that he is currently engaged in *independent and critical thinking* about the speaker's proposal.

Together with the succession of frowns the embodied display of independent thinking that accompanies the speaker's turn serves a number of functions on multiple interactional planes: first, it provides an online-feedback both for the current speaker and for the other co-participants on how the listener received the current speaker's argument. The publicly observable formation of the recipient's epistemic stance enables the other recipients to assess even during turn production how the perspectives of the individual participants, including their own, relate to each other. Obviously, this facilitates the coordination between the participants. With regard to the organization of turn-taking, the embodied display projects that the recipient is going to claim the floor. This is in line with Kendon's (1973) observation with regard to what he calls “long utterances” [e.g., “when people are exchanging points of view, (…) or exploring one another's knowledge of something,” p. 61]. Before the expectable end of the current speaker's turn, recipients regularly look away in order to signal readiness to take the turn. Producing thinking displays at turn-final position is thus a practice of self-selection (Sacks et al., 1974), which may be especially functional in multiparty conversations. In addition, the embodied display of critical thinking also prefigures what type of next action is to be expected: a multi-unit turn with which the plausibility of the speaker's position is challenged (line 151–155). It is thus a resource for the recipient to shape the emerging participation framework and to position himself as an attentive and aligning, yet autonomous thinker.

The data show that embodied displays of independent and critical thinking can also cause the current speaker to change his or her epistemic stance in the course of utterance production (cf. Kaukomaa et al., 2015 for emotional stances). This underlines the fact that recipient displays fulfill essential functions for the coordination between the participants.

#### Co-imagining and Co-exploring a Scenario Described by the Current Speaker

In contrast to the previous example, recipients may also embody that they co-imagine a scenario described by the current speaker and agree with his or her conclusion. These displays accompany the speaker's multi-unit turn. The following and final Extract 5 again stems from a discussion about the moral dilemma. Zarif suggested to return the trophy to the teacher and to organize a new competition (line 126–127), thus constructing a new scenario. When Yeliz expands Zarif's proposal, he accompanies her multi-unit turn with an embodied display of “doing thinking.”

**Extract 5:**
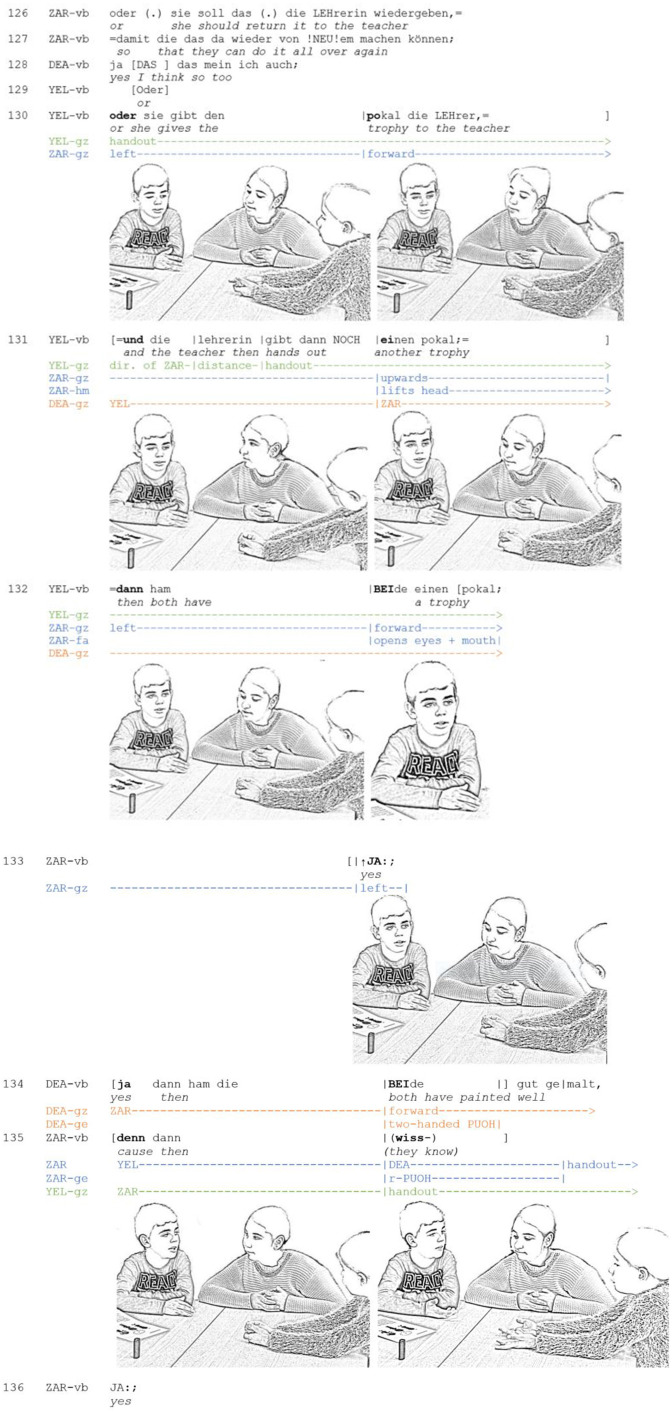
AM_G11_S_126-132 (DEA: Deana, YEL: Yeliz; ZAR: Zarif).

After Deana has agreed with Zarif's proposal, Yeliz produces a multi-unit turn (prefaced with “or,” line 129 and 130) in which she co-constructs but also slightly modifies Zarif's proposal. She first reformulates the first part of the scenario (line 130) and then adds a new idea to it: the teacher should award two trophies (line 131), with the consequence that both children win the competition (line 132). In this way, Yeliz makes the proposal a shared one. While she elaborates the scenario, Zarif reciprocates her posture and gaze behavior. First, Yeliz and Zarif adopt reciprocal postures: they lay their arms on the table and none of them reaches for the handout. Thus, both of them assume a rather inflexible posture. Likewise, both of them engage in imaginative gaze. While Yeliz formulates her proposal, she gazes in the direction of the handout. Yet she does not seem to focus something specific but rather gazes into the void, thus indicating a change in the direction of her (visual) attention. Zarif first shifts his gaze to the left, then in the empty space in front of him. This multimodal gestalt embodies that the scenario described by the current speaker gradually emerges before his mind's eye. Subsequently, his embodied display of *co-imagining and co-exploring a scenario* changes dynamically; each change is aligned with one element the semantic-pragmatic structure of the speaker's multi-unit turn. At end of the second turn-constructional unit, when Yeliz formulates the semantic core element of her alternative (“another/a second trophy”), Zarif lifts his head and shifts his gaze upwards, thus enacting that he has taken a new step in thought. In this way, he provides the other participants with a visible clue that he is following the speaker's idea step-by-step. In fact, this clue does not go unnoticed: Deana shifts her gaze toward Zarif (line 131). When Yeliz draws the consequence (line 132), Zarif raises his gaze again and lets it wander first to the left. Overlapping with the final element “both [.] a trophy,” he looks forward again and also opens his eyes and mouth—a multimodal gestalt that Heath et al. (2012, p. 217) refer to as “surprised mouth” –, giving the impression of having come to an insight. This is followed by a “↑YE:S” which is lengthened and also produced with a small pitch upstep. The prosodic design results in the fact that the “yes” is not only heard as a confirmation, but as an indication of an insight or “aha” moment. Together with the “yes” the facial expression thus embodies a change of state (cf. Mondada, 2011). Similar to “oh,” which is produced as a response to information of some kind and enacts a change in its producer's state of knowledge (Heritage, 1984), the embodied change of state described here serves not only to accept the prior talk as informative but also to register that the proposal developed by the speaker was persuasive. This way, it displays both *a change of state* and *a change of stance*.

Until now, the participants have not established mutual gaze. Instead, the recipient's ongoing and vivid wandering of the eyes served as an embodied display of co-imagining and co-exploring the scenario that the speaker currently describes. The participants have thus created a participation framework for joint imagination, within which each participant envisions and inspects the scenario. Only after the scenario was concluded and Zarif indicated a change of state and epistemic stance, the participants establish an F-formation: Zarif initiates mutual gaze with Yeliz, while Deana also looks at Zarif. Overlapping with Deana, Zarif then begins to elaborate Yeliz' idea by explicating the consequence in more detail. His palm presentation gesture (line 135) is produced in concert with Deana's two-handed palm presentation gesture. Both gestures serve to project a conclusion or concluding comment on the prior proposal (cf. Kendon, 2004, p. 270). By temporally aligning their gestures and directing them at each other, both participants mutually demonstrate to each other that they have reached a similar conclusion at the same time (Schönfelder and Heller, 2019). Although Zarif abandons his turn, it is clearly visible that both turns were designed to further co-construct the shared argument. The sequence is thus framed as a collaborative reasoning, in which hypothetical scenarios are jointly explored.

To summarize, within this process of co-constructing an argument, constant wandering of the eye is used by the recipient to demonstrate that he co-imagines the scenario described by the current speaker. Avoiding eye contact is important to convey that the recipient first envisions and explores the scenario on his own. Facial expressions serve to embody the progress of the thought process and a change of stance. The visible formation of the recipient's stance enables the other participants to observe “online” how an argument is received by one party. This enables them to anticipate at an early stage how the perspectives of those involved relate to each other. In the present case, this resulted in a concerted display of consent (line 134/135). Recipient displays of “doing thinking” thus have the potential to act as a catalyst for the decision-making processes of groups.

#### Summary

The multimodal gestalts of the recipients' embodied displays of thinking resembled those of the speakers. Table 2 summarizes the findings.

**Table 2:** Recipients' displays of “doing thinking.”

**Embodying independent and critical thinking**	thinking posture	imaginative gaze and wandering of the eyes	prefaced by frowns, emerging at the end of the speaker's multi-unit turn and lasting over the pause	solitary displacement/imagination; projecting disagreement and claim of the floor
**Co-imagining a scenario described by the current speaker**	inflexible posture	imaginative gaze and wandering of the eyes	accompanying the speaker's multi-unit turn	joint imagination; demonstrating agreement

On the whole, the embodiment of critical thinking is more prominent than that of co-imagining a scenario described by the current speaker: it contains not only a stylized thinking posture, but is also preceded by frowns. The greater prominence allows the recipient to point out problematic aspects while the speaker is still talking. In contrast, the lower salience of embodiments of co-imagining ensures that the attention of the current speaker is not distracted and that he can finish his multi-unit turn relatively undisturbed.

Both practices show that recipients are actively shaping the emerging interaction. The visible formation of the recipient's stance or knowledge enables both the current speaker and the other recipients to observe “online” how an argument is received by one party. In this way, recipient displays of “doing thinking” enable participants to anticipate different perspectives and to plan their next moves. They may thus act as a catalyst for the decision-making processes of groups.

## Discussion

Based on the seminal paper by Goodwin and Goodwin (1986) on thinking faces in word searches and recent conversational analytical studies on facial expressions (Peräkylä and Ruusuvuori, 2006; Ruusuvuori and Peräkylä, 2009; Kaukomaa et al., 2013, 2014, 2015) in face-to-face interactions, this paper investigated the epistemic and interactive functions of embodied displays of “doing thinking” in processes of argumentative decision-making. Using a corpus of video-recorded peer interactions, the study uncovered different practices of displaying thinking. The analysis showed that the embodied displays are context-sensitive and temporally coordinated with speech. Another finding is that thinking displays are not restricted to the face, but involve multiple resources. Among them are stylized thinking postures and imaginative gaze. Through marked contrasts between mobile and inflexible postures, the performers indicate the alignment of their attention to a world of thought, and vivid eye movements are used to evoke the impression of an ongoing thinking process. This shows that embodied displays of thinking are complex and highly dynamic multimodal gestalts.

The ways in which the embodied displays were used in interaction suggests that they should not be conceptualized as an external manifestation of internal processes. Regardless of whether they were produced by the speaker or the recipient, “doing thinking” was always organized as a public practice and multiparty activity: they were performed *for* the co-participants and served to mobilize their (visual) attention. In all examples presented here, it could be observed that the co-participants oriented toward the performer. In multiparty interactions, they are therefore an important resource for involving different parties in the activity-in-progress and shaping the emerging participation framework. In this respect, thinking displays fulfill essential *interactive functions* as assumed by Bavelas et al. (2014).

I shall argue, however, that embodied displays of “doing thinking” have other repercussions as well. By mobilizing the participants' attention, they create a space for the speaker to envision a hypothetical scenario and involve the participants in imagining potential consequences. Thinking displays thus seem to be ideally suited to constitute an *epistemic ecology for exploring ideas and collaborative reasoning*. This is of crucial importance for an exploratory framing of processes of argumentative decision-making.

Furthermore, investigating thinking displays in argumentative activities revealed that they also fulfill *epistemic functions*. When they were temporally aligned with lexical, syntactical, and/or morphological markers of epistemic modality, they were used to signal a thoughtful and tentative, independent yet cooperative, determined, affirmative or critical stance. Most importantly, due to their capacity to accompany longer stretches of talk and to change dynamically, they lend themselves to enact changes in the state of knowledge or stance. When recipients used thinking displays to mark their epistemic stance with regard to the speaker's position, this also implied showing that the other's ideas somehow affected their own thinking. This was particularly evident when recipients reciprocated the speaker's thinking displays (examples 1, 2, and 5). In this way, they emphasized the “jointness of emerging decisions” (Stevanovic et al., 2017).

The analysis is based on interaction data from children. However, there is little reason to assume that the practices reconstructed here are child-specific. For one thing, the analysis deliberately focused on older children who already have well-developed discursive skills. For another, the functions that the displays perform are also relevant in adult decision-making discourses. In this respect, the embodied displays are likely to prove a highly functional resource for adults as well.

This paper has presented multimodal and sequential analyses of embodied displays of “doing thinking” in a particular discursive practice. In order to fully understand thinking displays in naturally occurring interaction, future studies should examine their use in a range of different settings and discursive practices. Another area of future research concerns the acquisition of embodied argumentation. Assuming that the acquisition of discursive skills inherently involves the coordination of multimodal resources, the question arises as to how younger children come to use embodied thinking displays in different discursive activities. It can be expected that the conversational use of the “personal front” (Goffman, 1963) is a rather late achievement, because while multimodality is a resource, it is also a complex skill that itself needs to be acquired. This seems to apply especially to complex and dynamic thinking displays. Moreover, their interactive and epistemic functions are intricately interwoven with the discursive practice they are used for. Therefore, their acquisition should be closely related to the development of discursive skills. By describing the interactive and epistemic functions of thinking displays, the present article hopes to have created a first basis for investigating their acquisition.

## Data Availability Statement

The datasets presented in this article are not readily available because participants did not give written consent to share the data. Requests to access the datasets should be directed to Vivien Heller, vheller@uni-wuppertal.de.

## Ethics Statement

Ethical review and approval was not required for the study on human participants in accordance with the local legislation and institutional requirements. Written informed consent to participate in this study was provided by the participants' legal guardian/next of kin.

## Author Contributions

VH developed the theoretical framework, collected the data with her team, and carried out the empirical analysis.

## Conflict of Interest

The author declares that the research was conducted in the absence of any commercial or financial relationships that could be constructed as a potential conflict of interest.

## Footnotes

^1^The term “reasoning” is used here synonymously with that of conversational argumentation; “collaborative reasoning” refers to an exploratory mode of jointly developing and weighing arguments.

^2^Adobe Photoshop was used to create a sketch effect and to erase or change identifying features.
