# Supplementary material for: Embodied Displays of “Doing Thinking.” Epistemic and Interactive Functions of Thinking Displays in Children's Argumentative Activities
Source: Front Psychol. 2021 Feb 18;12:636671. doi: 10.3389/fpsyg.2021.636671 (PMC7935546; doi:10.3389/fpsyg.2021.636671)
Supplement: Supplementary file 1 [file Table_1.docx]

Supplementary Material

GAT 2 transcription conventions

(Selting et al., 2009; for the English translation cf. Couper-Kuhlen and Barth-Weingarten, 2011)

Sequential structure

[ ] overlap and simultaneous talk

[ ]

= immediate continuation with a new turn or segment, latching

In- and outbreaths

°h / h° in-/outbreaths of appr. 0.2-0.5 sec. duration

°hh / hh° in-/outbreaths of appr. 0.5-0.8 sec. duration
°hhh / hhh° in-/ outbreaths of appr. 0.8-1.0 sec. duration

Pauses

(.) micro pause, estimated, up to 0.2 sec. duration appr.

(-) short estimated pause of appr. 0.2-0.5 sec. duration

(--) intermediary estimated pause of appr. 0.5-0.8 sec. duration

(---) longer estimated pause of appr. 0.8-1.0 sec. duration

(0.5)/(2.0) measured pause of appr. 0.5/2.0 sec. duration

Segmental conventions

and_uh clitizations within units

uh, uhm, etc. hesitation markers, so-called "filled pauses"

: lengthening, by about 0.2-0.5 sec.

:: lengthening, by about 0.5-0.8 sec.

::: lengthening, by about 0.8-1.0 sec.

Laughter

haha, hehe, hihi syllabic laughter

((laughs)) description of laughter and crying

<<laughing> > laughter particles accompanying speech with indication of scope

Continuers

hm, yes, no, yeah monosyllabic tokens

hm_hm, ye_es, bi-syllabic tokens

ʔhmʔhm with glottal closure, often negating

Accentuation

SYLlable focus accent

sYllable secondary accent

!SYL!lable extra strong accent

Final pitch movements of intonation phrases

? rising to high

, rising to mid

– level

; falling to mid

. falling to low

Loudness with scope, other conventions

<<f> > forte, loud

<<ff> > fortissimo, very loud

<<p> > piano, soft

<<pp> > pianissimo, very soft

<<surprised> > interpretive comment with indication of scope

(xxx), (xxx xxx) one or two unintelligible syllables

(may i) assumed wording

Multimodal transcription conventions

The multimodal transcription of embodied behavior is based on the following conventions (adapted with changes from Mondada, 2019):

PAR-vb verbal action of participant PAR

PAR-fa facial action of participant PAR

PAR-gz gaze behavior of participant PAR

PAR-ge gesture of participant PAR

PAR-po posture of participant PAR

PAR-hm head movement of participant PAR

---> embodied behavior is continued across subsequent lines

| | beginning and end of embodied behavior; if omitted, the behavior starts

or ends before or after the transcript

**bold face** the exact moment in which the screenshot has been taken, precisely

aligned with the co-occurring speech syllable

References

Couper-Kuhlen, E., and Barth-Weingarten, D. (2011). A system for transcribing talk-in-interaction: GAT 2 translated and adapted for English. *Gesprächsforschung. Online-Zeitschrift zur verbalen Interaktion* 12, 1–51.

Mondada, L. (2019). Conventions for multimodal transcription. Available at: https://www.lorenzamondada.net/multimodal-transcription [Accessed July 20, 2020].

Selting, M., Auer, P., Barth-Weingarten, D., Bergmann, J., Bergmann, P., Birkner, K., et al. (2009). Gesprächsanalytisches Transkriptionssystem 2 (GAT 2). *Gesprächsforschung – Online-Zeitschrift zur verbalen Interaktion* 10, 353–402.
